# Tau oligomers mediate α-synuclein toxicity and can be targeted by immunotherapy

**DOI:** 10.1186/s13024-018-0245-9

**Published:** 2018-03-15

**Authors:** Julia E. Gerson, Kathleen M. Farmer, Natalie Henson, Diana L. Castillo-Carranza, Mariana Carretero Murillo, Urmi Sengupta, Alan Barrett, Rakez Kayed

**Affiliations:** 1 Departments of Neurology, Neuroscience and Cell Biology, University of Texas Medical Branch, Galveston, TX 77555 USA; 2 Sealy Center for Vaccine Development, University of Texas Medical Branch, Galveston, TX 77555 USA

**Keywords:** Tau, Immunotherapy, Oligomers, α-synuclein, Synucleinopathies, Neurodegeneration

## Abstract

**Background:**

We have evaluated the efficacy of targeting the toxic, oligomeric form of tau protein by passive immunotherapy in a mouse model of synucleinopathy. Parkinson’s disease and Lewy body dementia are two of the most common neurodegenerative disorders and are primarily characterized by the accumulation of α-synuclein in Lewy bodies. However, evidence shows that smaller, oligomeric aggregates are likely the most toxic form of the protein. Moreover, a large body of research suggests that α-synuclein interacts with tau in disease and may act in a synergistic mechanism, implicating tau oligomers as a potential therapeutic target.

**Methods:**

We treated seven-month-old mice overexpressing mutated α-synuclein (A53T mice) with tau oligomer-specific monoclonal antibody (TOMA) and a control antibody and assessed both behavioral and pathological phenotypes.

**Results:**

We found that A53T mice treated with TOMA were protected from cognitive and motor deficits two weeks after a single injection. Levels of toxic tau oligomers were specifically decreased in the brains of TOMA-treated mice. Tau oligomer depletion also protected against dopamine and synaptic protein loss.

**Conclusion:**

These results indicate that targeting tau oligomers is beneficial for a mouse model of synucleinopathy and may be a viable therapeutic strategy for treating diseases in which tau and α-synuclein have a synergistic toxicity.

## Background

Parkinson’s disease (PD) and Lewy body dementia (LBD), behind Alzheimer’s disease (AD), are the most common neurodegenerative disorders with no effective therapies targeting the cause of disease. Passive immunotherapy is a promising mode of treatment, with a recent anti-amyloid oligomer antibody showing promising results [1]. After years of failure targeting amyloid-β protein, new promising approaches for AD targeting tau toxicity are coming to the forefront [2].

The pathological hallmarks of PD are cytoplasmic inclusions called Lewy bodies (LB), comprised primarily of α-synuclein, along with hyperphosphorylated tau and other sequestered proteins [3–8] in dopaminergic neurons. However, the importance of LB to the neurotoxicity in disease has been questioned [9–13]. A number of studies have shown that oligomeric α-synuclein is the toxic species, rather than fibrils comprising LBs [14–17], and that α-synuclein oligomers may be the most effective therapeutic target [18]. In spite of the clear prevalence of α-synuclein pathology in disease, one of the greatest genetic risk factors for PD is tau, the role of which is understudied and poorly understood [19]. Phosphorylated tau aggregates have been reported in numerous synucleinopathy mouse models [20–25], suggesting a possible synergistic interaction between α-synuclein and tau in mediating neurodegeneration in PD, as α-synuclein may increase tau aggregation [23, 26–29] and tau may have a similar effect on α-synuclein. While neurofibrillary tangles (NFTs) characterize tauopathies and are not correlative of synucleinopathies, recent studies suggest that intermediate forms of tau—tau oligomers—that form prior to or independently of NFTs, are the true toxic species in disease and the optimum targets for anti-tau therapies [30–35].

Tau and α-synuclein have been shown to interact in disease by multiple different groups. Large genome-wide association studies have revealed an increased risk for PD and α-synuclein toxicity associated with changes to the gene encoding tau [36, 37]. Additionally, the proteins have been found to co-exist in disease and α-synuclein aggregation has been demonstrated to cause an increase in tau fibril levels [26, 38]. Increasing evidence also suggests that comorbidity of AD pathology in synucleinopathies is common [39]. Moreover, decreasing tau in a synucleinopathy mouse model has been shown to be protective [40]. We have shown that, unlike fibrils, oligomeric α-synuclein can seed the formation of tau oligomers [27]. Given the presence of tau oligomers in PD and LBD human brains [41], this highlights their importance in synucleinopathies. Thus, oligomeric tau may represent a new therapeutic target for synucleinopathies.

A53T mice overexpress a mutation in α -synuclein that leads to PD in humans and have been shown to have both tau and α-synuclein aggregates [42], resulting in motor and cognitive deficits [43, 44]. Therefore, we chose to specifically target oligomeric tau by passive immunotherapy in A53T mice with tau oligomer-specific monoclonal antibody (TOMA) to evaluate the efficacy of tau oligomers as a novel drug target for the treatment of synucleinopathies.

## Methods

### Preparation of brain homogenate

Frozen frontal cortical brain tissue from PD subjects was received in blocks from Oregon Health & Science University. Tissues used for research were approved by the Institutional Ethics Committee. Brains were homogenized in PBS with a protease inhibitor cocktail (catalog no. 11836145001; Roche Applied Science, Indianapolis, IN, USA), using a 1:3 dilution of tissue: PBS (*w*/*v*). Samples were centrifuged at 10,000 rpm for 10 min at 4 °C. Supernatants were aliquoted, snap-frozen, and stored at − 80 °C until use. For insoluble tau fractions, pellets were resuspended in 10% Sarkosyl in PBS, vortexed, and sonicated, then centrifuged for 30 min at 100,000 x g at 4 °C.

### Non-denaturing filter trap assay

Tau, amyloid-β and α-synuclein oligomers and tau monomer and fibrils were prepared as previously described [45]. Filter Trap assay was performed to detect tau oligomers with conformational antibodies in the absence of reducing agents. Briefly, 1 μg of oligomeric tau was applied to nitrocellulose membranes, previously saturated with TBS-T, through the use of a vacuum-based bio-slot apparatus. Then, membranes were blocked with 10% nonfat milk in TBS-T overnight at 4 °C. The next day, membranes were probed with the oligomer-specific tau antibodies, T22 (1:250) and TOMA (1:250) as well as total tau antibody, tau5 (1:10000), or α-synuclein antibody, 4D6 (1:5000), or amyloid-β antibody, 6E10 (1:5000), diluted in 5% nonfat milk for 1 h at RT. Membranes were then washed three times with TBS-T and incubated with horseradish peroxidase-conjugated IgG anti-rabbit (1:5000) and anti-mouse (1:5000) secondary antibodies as appropriate. Membranes were washed again three times in TBS-T and ECL plus (GE Healthcare) was used for signal detection.

### Animals

This study was conducted in a facility approved by the American Association for the Accreditation of Laboratory Animal Care, and all experiments were performed in accordance with the National Institutes of Health *Guide for the Care and Use of Laboratory Animals* and approved by the Institutional Animal Care and Use Committee of the University of Texas Medical Branch (UTMB). For immunotherapy experiments, both male and female animals were balanced in each group to determine whether sex differences were present. A53T mice (The Jackson laboratory; B6;C3-Tg(Prnp-SNCA*A53T)83Vle/J) and nontransgenic littermate (WT) animals were bred at UTMB and were raised free of enrichment following weaning to prevent any effect on behavioral test performance. Mice were housed at the UTMB animal care facility and maintained according to U.S. Department of Agriculture standards (12 h light/dark cycle with food and water available ad libitum).

### Experimental design and statistical analysis

Treatment groups were assigned at random using a random number generator. Two-tailed power analysis using Cohen’s Power Table when α = 0.05 was conducted to determine the appropriate sample size for each immunotherapy group based on the means and standard deviations from a pilot immunotherapy study in A53T mice. It was determined that a sample size of 16 would allow us to detect a difference in discrimination index of 0.45 between groups on the novel object recognition task 80% of the time. Additional animals were included in A53T groups to protect against any unforeseen attrition or sex-dependent effects. Half of the mice in each group were maintained after behavioral analysis to assess mortality using a Kaplan-Meier survival curve, analyzed by the Log-rank (Mantel Cox) test with a Bonferroni adjustment for multiple comparisons (threshold for significance *p* < 0.025).

### Immunization

Seven-month-old homozygous A53T mice were given intravenous injections of 120 μL per animal of either of 1 mg/mL TOMA (*n* = 17), or anti-Rhodamine antibody (IgG, nonspecific; catalog #GTX29093; Genetex; *n* = 23) for the control treatment group (Ctrl IgG). Wildtype littermates were given 120 μL of saline injected intravenously (*n* = 16). Injections were repeated a second time on seven-month old homozygous A53T mice treated with either TOMA (*n* = 5) or saline (*n* = 4) in order to confirm that differences were not due to toxicity of non-specific IgG antibody in Ctrl IgG mice. For intravenous injections, mice were placed in a restrainer (Braintree Scientific), and an inch of the tail was shaved and placed in warm water to dilate veins. Mice were then injected via the lateral tail vein, returned to home cages, and kept under observation.

### Behavioral analyses

Prior to performing behavioral tasks, mice were habituated to frequent experimenter handling. All tasks were scored and analyzed by experimenters blinded to treatment group. To determine the effect of immunotherapy on behavioral deficits, the cognition and motor phenotype of A53T mice was evaluated using the NOR, rotarod, footprint and nesting tests. The NOR test was performed 2 weeks after immunization, followed by the two motor tasks (Fig. 2).

### Novel object recognition (NOR)

The novel object recognition task (NOR) utilizes the natural tendency of rodents to preferentially explore novel objects and environments over those that are familiar. A53T mice have been shown to exhibit memory deficits which may be detected by NOR starting at 6 months of age [44, 46]. Mice were habituated to the NOR task and allowed to freely explore the white open-field arena (55 cm in diameter; 60 cm in height) for 15 min on the first day. The next day, mice were placed in the arena for the training phase with two identical objects, either spheres or cubes, and allowed to explore for 15 min. On the third day, mice were placed again in the arena for 15 min with one familiar object previously explored in the training phase and one novel object differing in color and shape, but sharing a common size and volume. After each trial, the apparatus was thoroughly cleaned using 70% Ethanol and allowed to dry prior to placement of a new mouse. Trials were recorded and time spent exploring each object was measured using ANY-Maze software. Exploration was defined by head orientation within 2 cm of the object or physical contact with the object. The percentage of total time spent exploring the familiar object versus the novel object was measured. In order to control for any differences in exploratory behavior, the discrimination index was also calculated as the time spent exploring the familiar object subtracted from the time spent exploring the novel object, divided by the total time spent exploring both objects. Thus, a higher discrimination index is indicative of functioning object discrimination and improved cognitive function. Object exploration data were analyzed using Graph Pad Prism 5.04 sofware by one-way analysis of variance (ANOVA) and Tukey’s post-hoc test.

### Rotarod test

A53T mice do not demonstrate gross motor deficits until around nine to 12 months of age [44, 47]. Rotarod measures the ability of a rodent to maintain balance on a rotating rod (diameter 3.2 cm) and may be used to evaluate both motor coordination and hyperactivity. Mice were first habituated in one session of four trials to reach a baseline level of performance. The next day, mice were tested in one session of four trials (Rotarod meter; Stoelting). Five mice were placed onto the rod, one per testing station. The speed started at 4 rpm and accelerated at 0.1 rpm/s. The latency to fall from the rotating rod was determined. Latency significantly higher than that of wildtype was determined to be indicative of a hyperactivity phenotype. Data were analyzed using Graph Pad Prism 5.04 sofware by one-way analysis of variance (ANOVA) and Tukey’s post-hoc test.

### Footprint test

A53T mice have been shown to have gait alterations similarly to what is seen in PD disease patients clinically [44, 48]. The footprint test was used to compare the gait of A53T transgenic mice in each treatment group with that of wild-type control mice. Mice were first trained to walk along a 50-cm-long, 10-cm-wide runway (with 10-cm-high walls) into an enclosed box containing fruit cereal. All mice had four training trials. To obtain footprints, hindfeet and forefeet of each mouse were coated with blue and red nontoxic paints, respectively. One day following training, mice were allowed to complete a testing trial. Any mouse that did not walk continuously along path was given another trial. A fresh sheet of white paper was placed on the floor of the runway for each run. Footprints were analyzed for different gait parameters measured in centimeters as has been previously described [49], including stride length (average forward distance between each stride), hindpaw width and forepaw width (average distance between left and right hind and front footprints, respectively), and front footprint /hind footprint overlap for uniformity of steps (a value of zero is recorded when the center of the hind and front footprints overlap and the distance between the two centers was recorded for those which did not overlap). For each step parameter, six to eight values were measured from each run, excluding footprints made at the beginning and the end of the run to avoid inaccurate measurements during initiation and completion of movement. The mean value of each set of measurements was compared between groups by one-way ANOVA and Tukey’s post-hoc test using Graph Pad Prism 5.04 software.

### Nesting test

The nesting task is designed to utilize the innate ability of both male and female mice to build nests for heat, shelter, and reproduction and has been shown to be affected by very minor sensorimotor deficits [50]. A53T mice begin to show minor deficits that can be detected by nesting behavior as young as two to 3 months of age, prior to any gross locomotor phenotype [44, 51]. The nesting task was completed as previously described [44, 50]. Two hours prior to the dark phase, each mouse was placed in an individual testing cage identical to home cage and given a single cotton nestlet. At 1 h and 24 h after placement, nesting quality was determined by two independent researchers blinded to treatment group based on previously defined criteria described as follows; 1: nestlet not visibly touched; 2: nestlet partially torn but at least 50% remaining untouched; 3: 50-90% of nestlet shredded but scattered distribution throughout the cage; 4: at least 90% of nestlet is shredded and a compact, flat nest can be identified; 5: a nearly perfect nest with over 90% shredded and walls of the nest higher than the mouse’s body. Nesting scores were averaged and analyzed using Graph Pad Prism 5.04 software by the Kruskal-Wallis non-parametric ANOVA and Tukey’s post-hoc test.

### Tissue collection

To evaluate the pathological presence of oligomeric tau in synucleinopathy mice, brain samples were collected from A53T [51] and non-transgenic control mice at different ages. For the immunotherapy experiment, A53T mice from each group were randomly selected following completion of behavioral testing (*n* = 7/group), as well as a second set of treated mice (*n* = 4-5/treatment group) not tested behaviorally for confirmation. Mice were euthanized with CO_2_ and brains were collected for analysis. The right hemisphere of each brain was snap-frozen and homogenized for biochemical analysis. The left hemisphere of each brain was embedded in optimal cutting temperature medium (OCT), rapid frozen and sectioned. All sections (7 μm) were processed simultaneously under the same conditions.

### Immunohistochemistry

Sections were fixed in methanol at − 20 °C, permeabilized with 0.5% Triton-X, incubated with 3% hydrogen peroxide to quench endogenous peroxidases and blocked in 5% goat serum for 1 h. Sections were then incubated overnight at 4 °C with either T22 for tau oligomers (1:200) or tyrosine hydroxylase antibody (Millipore) for dopamine (1:200). The following day, sections were washed in PBS three times for 10 min each and incubated with biotinylated goat anti-rabbit IgG (1:200; Vector Laboratories) for 1 h. Sections were then washed three times for 10 min each in PBS and processed using an ABC reagent kit (Vector Laboratories) and visualized with DAB substrate (Vector Laboratories) according to the manufacturer’s recommendations. Lastly, sections were counterstained with hematoxylin (Vector Laboratories) for nuclear staining and mounted. To quantify TH-positive cells, we acquired bright field images using a Nikon Multizoom AZ100 microscope equipped with a Nikon DS-2 M color CCD camera (Nikon Instruments Inc.). Three sections per mouse (*n* = 6-8 per treatment group) were imaged for quantification. Four visual fields from the olfactory bulb of each section were used to quantify TH-positive cell bodies using the ImageJ plugin, counting cell. Results were analyzed by one-way ANOVA with the Bonferroni post hoc test.

### Immunofluorescence

Sections used for immunofluorescence were fixed in 4% paraformaldehyde, permeabilized with 0.5% Triton-X and blocked in 5% goat serum for 1 h. Prior to permeabilization step, sections evaluated for Proteinase K (PK) sensitivity were treated with 10 μg/ml PK (Sigma Aldrich, cat. #P2308) for 10 min. Sections labeled with antibodies raised in mouse went through a second blocking step with unconjugated Fab anti-mouse IgG (40 μg/ml; Jackson Immunoresearch) for 1 h to block endogenous IgG immediately following blocking with serum. Sections were then incubated overnight at 4 °C with either tau oligomer-specific polyclonal antibody T22 (1:250), α-synuclein oligomer-specific antibody Syn33 (1:100), total α-synuclein antibody 4D6 (1:400; Abcam, cat. #ab1903), Lewy body antibody LB509 (1:300; Abcam, cat. #ab27766) or GFAP (1:300; Covance, cat. #SM1-224). The following day, sections were washed in PBS three times for 10 min each and incubated with goat anti-rabbit IgG Alexa-488 and anti-mouse IgG Alexa-568 (1:500; Invitrogen) for 1 h. Sections were then washed in PBS three times for 10 min each and incubated with DAPI (Invitrogen) to label nuclei for 5 min at room temperature, washed three times for 5 min each, and were mounted with Fluoromount G (Southern Biotech). The sections were examined using a Nikon A1R MP Laser scanning microscope. LB509 labeled sections were analyzed using Cell Counter in Image-J (National Institutes of Health) for number of Lewy body-like structures (*n* = 6-8/group). For all other antibodies, the total level of fluorescence was measured for each randomly selected cell using Image-J, as well as the level of background from three different surrounding regions without fluorescence (n = 6-8/group). To correct the level of fluorescence for background and cell size, the background multiplied by the area of the region was subtracted from the total fluorescence. Corrected fluorescence was equilibrated to nuclear fluorescence measured by DAPI to control for differences in cell density. Results were analyzed by one-way ANOVA with the Bonferroni post hoc test.

### Western blot analysis

PBS soluble fractions of brain extracts containing 20 μg of total protein were loaded (without boiling) on precast NuPAGE 4-12% Bis-Tris gels (Invitrogen) for SDS-PAGE analysis. Gels were subsequently transferred onto nitrocellulose membranes and blocked overnight at 4 °C with 10% nonfat dry milk. Membranes were then probed for 1 h at room temperature with T22 (1:250), Tau5 (1:2000), F8H7 (1:200), Syn33 (1:500), α-synuclein phospho S129 (1:1000; Abcam cat #ab59264), tyrosine hydroxylase (1:1000), synapsin 1 (1:1000), synaptophysin (1:500), and β-actin (1:2000) diluted in 5% nonfat dry milk. T22, tyrosine hydroxylase, α-synuclein phospho S129, Syn33 and Synapsin 1 were detected with an HRP-conjugated anti-rabbit IgG (1:3000, GE Healthcare), while Tau5, synaptophysin, F8H7, and β-actin were detected with an HRP-conjugated anti-mouse IgG (1:3000, GE Healthcare), diluted in 5% milk. ECL (Advansta) was used to visualize the bands, which were normalized to corresponding β-actin levels. Densitometric analysis was performed using ImageJ software (National Institutes of Health). Analyses were completed in triplicate and analyzed by one-way ANOVA with the Bonferroni post hoc test.

### ELISA

For ELISA we used Nunc immobilizer amino modules, 96 well plates (Thermo Fisher Scientific). Plates were coated with 60 ng/well of brain homogenate using 0.1 M sodium bicarbonate, pH 9.6, as coating buffer, followed by overnight incubation at 4 °C, plates were washed 3 times with Tris-buffered saline (TBST) with low Tween (0.01%), then blocked for 3 h at room temperature with 10% nonfat dry milk in TBST. The plates were then washed with TBST. Primary antibodies, T22 (1:250), Syn33 and 4D6 (1:1000) diluted in 5% nonfat dry milk in TBST were then added and allowed to react for 1 h at room temperature. The plates were then washed 3 times with TBST and 100 μl of horseradish peroxidase-conjugated anti-rabbit and anti-mouse IgG (1:3000; GE Healthcare Life Sciences, Pittsburgh, PA) were added, followed by incubation for 1 h at room temperature. Finally, plates were washed three times with TBST and developed with 3,3′,5,5′,-tetramethylbenzidine (TMB-1) substrate from DAKO (Carpinteria, CA). The reaction was stopped with 100 μl 1 M HCl and absorbances were read at 450 nm using a POLARstar OMEGA plate reader (BMG Labtechnologies, Melbourne, Australia). All measurements were performed in triplicate and analyzed by one-way ANOVA with the Bonferroni post hoc test.

## Results

### Elevated levels of tau oligomers in α-synuclein mouse models and PD

**Fig. 1:**
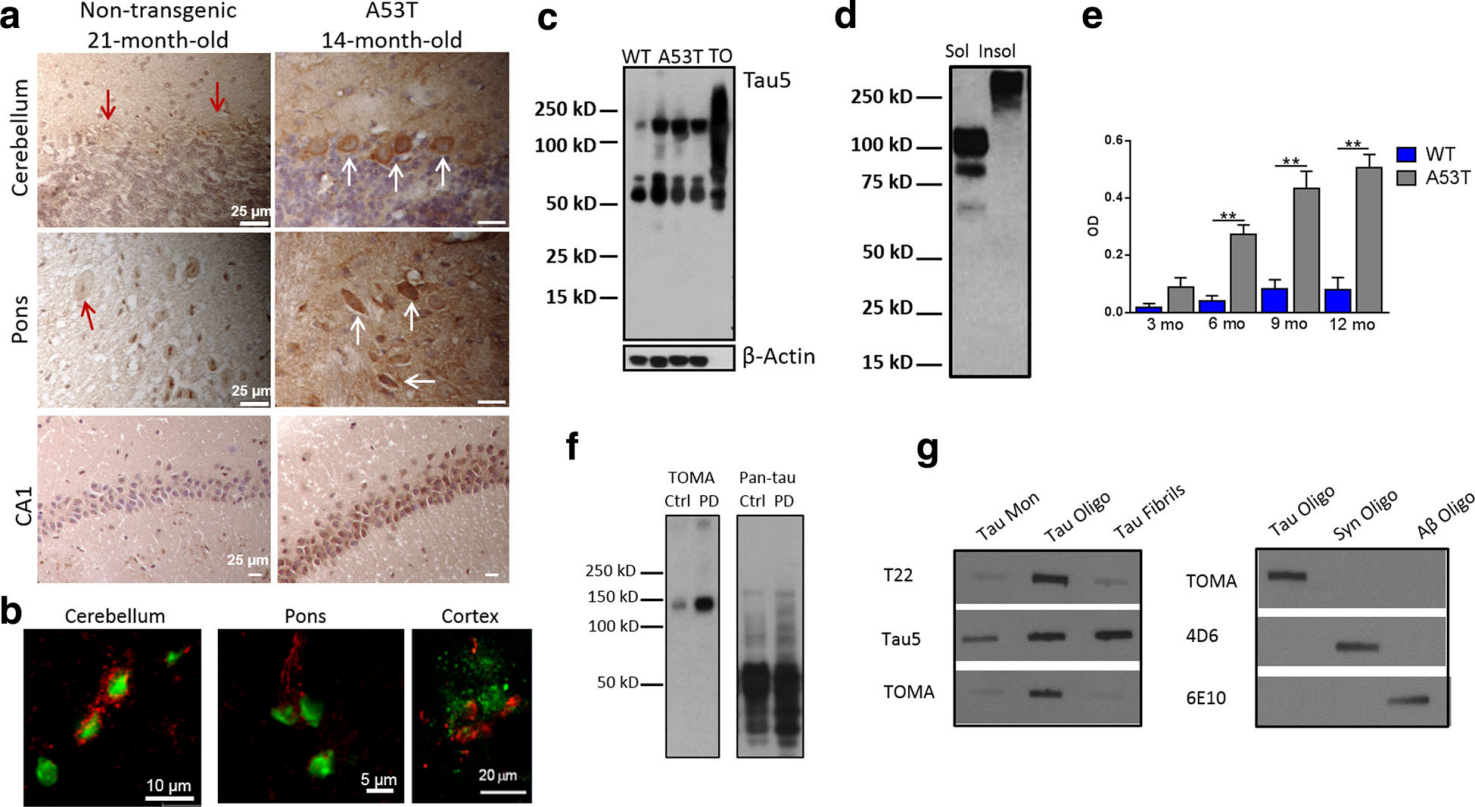
Tau oligomers are associated with α-synuclein toxicity. **a** Tau oligomers in A53T mice detected with T22 are elevated compared to non-transgenic controls. **b** Tau oligomers with T22 (red) are found in the vicinity of α-synuclein oligomers (green) in A53T mouse brains. **c** Western blot with Tau5 shows elevated oligomeric tau in A53T mice compared to non-transgenic (WT) controls. Recombinant tau oligomers (TO). **d** Western blot with TOMA shows detection of tau oligomers in PBS soluble (sol) and Sarkosyl soluble (insol) A53T mouse brain. **e** ELISA analysis of the PBS soluble fraction from whole brain shows that tau oligomer levels in the A53T mouse model are elevated compared to wild-type mice and increase with age. **f** Elevated levels of tau oligomers are detected in the PBS soluble brain homogenate of PD patients by Western blot with TOMA and a pan-tau antibody. **g** Non-denaturing filter trap assay demonstrates specificity of tau oligomer-specific antibodies. **p < 0.01

We previously characterized the presence of elevated tau oligomeric species in synucleinopathy human brains. The presence of phosphorylated and aggregated tau has been identified in synucleinopathy mouse model, Prnp-SNCA*A53T, and here we show that tau oligomers are also increased in the brains of these mice **(**Fig. 1a-e**)**. It is important to note that these animals are not designed to overexpress tau, yet, using the tau oligomer-specific antibody, T22, we found increased levels of oligomeric tau in the brains of these animals. Tau oligomers were elevated in brain regions important for cognitive function (hippocampus), as well as motor areas including pons and cerebellum. The molecular weight of these SDS stable oligomers is similar to those found in mouse models of tauopathy [30, 31, 52]. Additionally, tau oligomers were detected at elevated levels in the brain of PD patients by Western blot with TOMA **(**Fig. 1f**)**, as previously shown. Specificity of previously characterized tau oligomer-specific antibodies [53–55] is further demonstrated by non-denaturing filter trap assay (Fig. 1g).

### TOMA protects against behavioral deficits in A53T mice

**Fig. 2:**
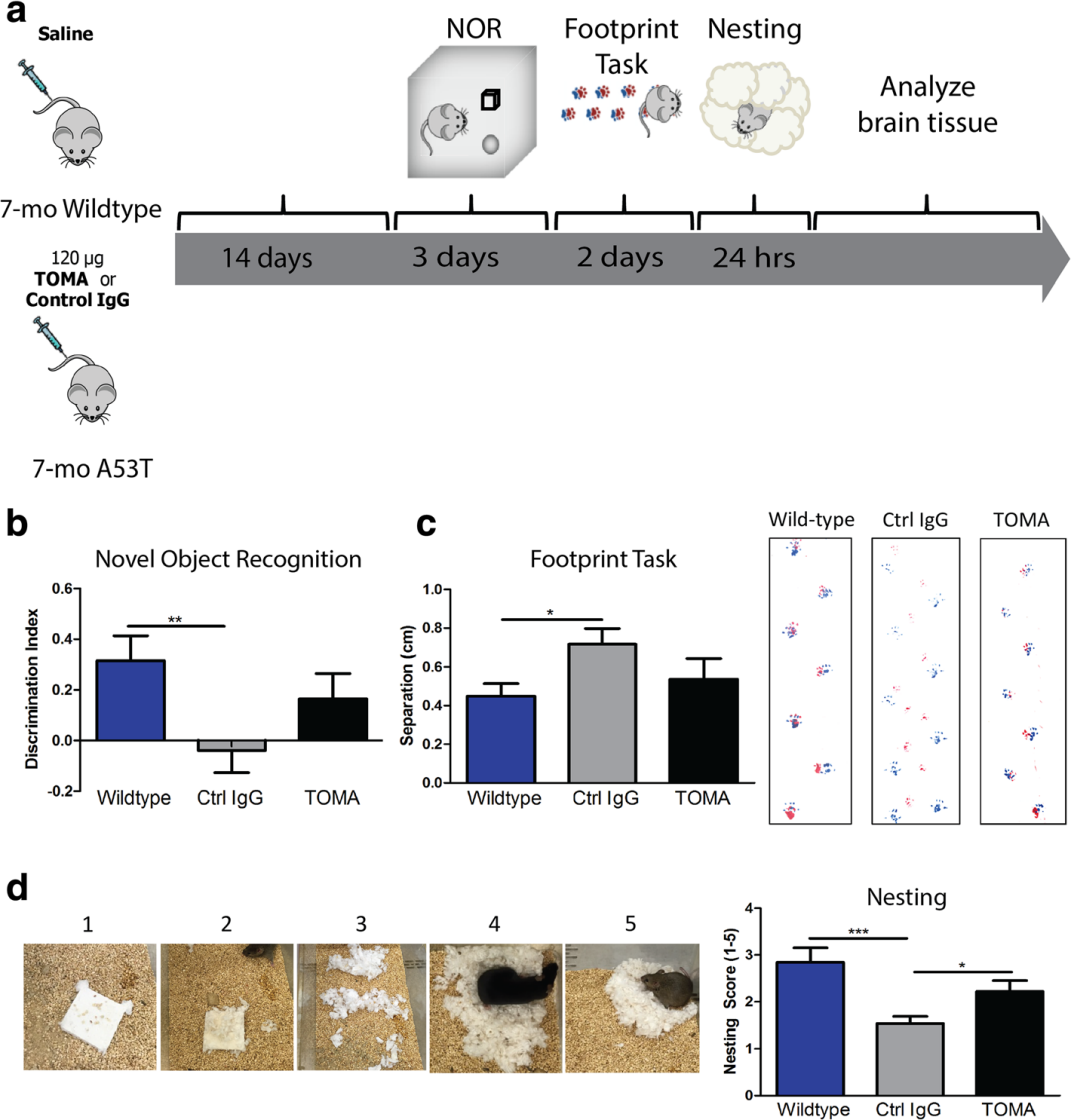
TOMA protects A53T mice from behavioral deficits. **a** Timeline for experimental design. **b** TOMA-treated animals are protected against memory deficits in the NOR. Control IgG-treated A53T mice have significantly lower discrimination index scores on the NOR when compared to wildtype mice (*p* = 0.0099) while TOMA-treated mice do not perform significantly differently than wildtype. **c** TOMA treatment protects against gait abnormalities in the footprint task. Control-IgG-treated mice have significantly greater separation between front and hind footprints when compared to wildtype (*p* = 0.048), while TOMA-treated mice do not perform differently than wildtype. **d** Control IgG antibody have significantly lower scores for nesting quality when compared to Wild-type controls (*p* = 0.0001) and TOMA-treated mice (*p* = 0.028), while TOMA-treated mice display nesting quality scores similar to wildtype. TOMA (*n* = 17), Ctrl IgG (*n* = 23), Wildtype (*n* = 16)

IV injection of TOMA has been shown to specifically modulate tau oligomers and reverse the phenotypes in three different animal models of tauopathy and Alzheimer’s disease without any side effects [53–55]. To test whether tau oligomer immunotherapy can protect against deficits in a synucleinopathy mouse model, we injected 7-month-old A53T mice with either TOMA or a control IgG and compared them to wild-type littermate controls injected with saline. Two weeks after injection, we conducted behavioral tasks for cognition and motor behavior, as summarized (Fig. 2a). A53T mice showed memory deficits beginning at 6 months of age [44, 46]. Using the NOR task, we found that A53T mice injected with control IgG antibody had significantly lower discrimination index scores when compared to wild-type animals (*p* = 0.0099), indicating a cognitive function deficit, based on the inability to discriminate between novel and familiar objects. A53T mice injected with TOMA showed no significant differences from wildtype or control IgG (Fig. 2b**)**. No differences were detected between wild-type animals or either A53T treatment group in the rotarod task (data not shown), indicating that gross motor impairment was not measurable at this age. Thus we utilized additional motor tasks that provide more sensitivity at younger ages in A53T mice. We performed the footprint task in order to measure differences in gait seen in the different treatment groups. We found no significant differences on all but one of the parameters. In the measure of front and hind paw overlap, however, only the control IgG-treated A53T mice showed greater distance between the front and hind paws when compared to Wild-type (*p* = 0.048), while TOMA-treated mice were no different than Wild-type controls and were not significantly different from control IgG mice **(**Fig. 2c). In order to detect more sensitive differences in motor behavior that can be seen starting around 2 months of age [44], we performed the nesting test. Representative images for each score on the task were recorded **(**Fig. 2d). One hour after receiving a cotton nestlet, A53T mice treated with control IgG antibody had significantly lower scores for nesting quality when compared to Wild-type controls (*p* = 0.0001) and TOMA-treated mice (*p* = 0.028), while TOMA-treated mice showed no differences from Wild-type **(**Fig. 2d).

### TOMA specifically lowers levels of tau oligomers

**Fig. 3:**
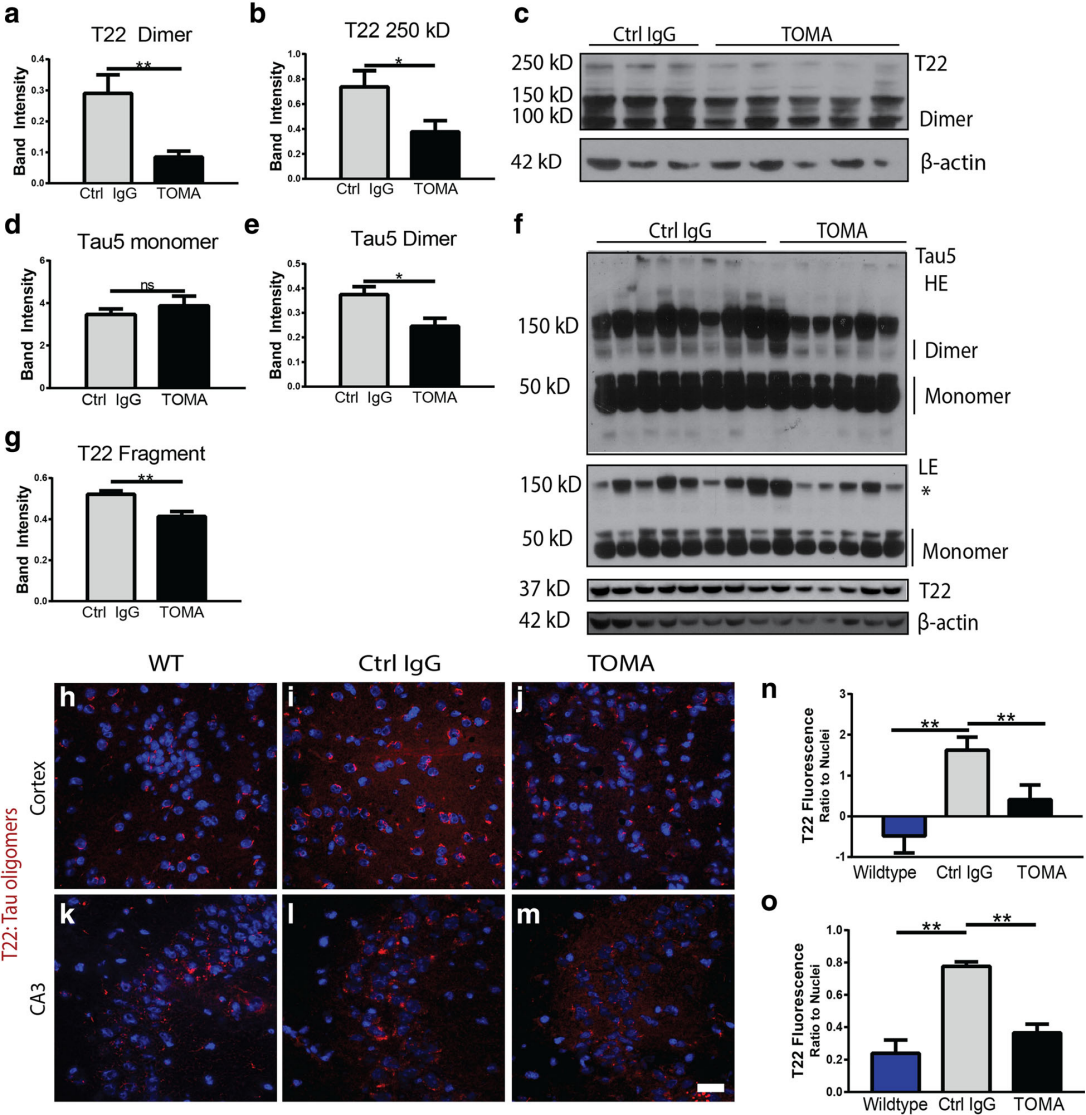
Levels of tau oligomers are specifically reduced in A53T mice treated with TOMA. **a**-**c** Western blot of PBS soluble total brain homogenate with tau oligomer-specific antibody, T22, showed that levels of tau dimer (low molecular weight oligomers) were significantly lowered in TOMA-treated mice compared to control IgG-treated mice (A; *p* = 0.009), as well as 250 kD (high molecular weight) tau oligomers (B; *p* = 0.036). **d**-**f** Tau monomer levels measured by Western blot with Tau5 were unaffected by treatment, while tau dimer was significantly decreased with TOMA. LE (low exposure) HE (high exposure). *Denotes non-specific band. **f**-**g** Western blot of the PBS soluble fraction of whole brain homogenate with T22 for tau oligomers showed that TOMA-treated mice had significantly lower levels of a 37 kD fragment compared to Control IgG (*p* = 0.003). **h**-**m** Immunofluorescence imaging detects significantly lower levels of tau oligomers in cortex (**h**-**j**, **n**) and hippocampi (**k**-**m**, **o**) of TOMA-treated mice. Control IgG-treated mice had significantly higher levels of T22 fluorescence intensity as a ratio to nuclei when compared to wildtype mice (*p* = 0.004) and TOMA-treated A53T mice (*p* = 0.009) in the cortex, as well as significantly higher levels in the hippocampus compared to wildtype mice (*p* = 0.001) and TOMA-treated A53T mice (*p* = 0.0044). Scale bar 50 μm

Immediately following the completion of behavioral examination, mice were euthanized and brains were extracted for analysis. By Western blot of PBS-soluble whole brain homogenate with T22 antibody, we found that TOMA-treated A53T mice had significantly lower levels of both low (*p* = 0.009) and high molecular weight (*p* = 0.036) tau oligomers when compared to A53T mice treated with a control IgG antibody (Fig. 3a-c). Using a pan-tau antibody, Tau5, that recognizes the various aggregation states of the protein, we showed that TOMA treatment did not decrease levels of the functional, monomeric form of tau (Fig. 3d, f**),** while tau dimer was significantly decreased (*p* = 0.02; Fig. 3e-f). Levels of a 37kD tau fragment associated with neurodegeneration [56] that is recognized by T22 antibody were also significantly decreased in TOMA-treated mice when compared to Control-IgG (*p* = 0.003; Fig. 3f-g). Immunofluorescence analysis with T22 for tau oligomers (Fig. 3h-o) showed significantly decreased tau oligomers in cortex and hippocampus of TOMA-treated mice when compared to Control IgG (*p* = 0.0089). Wildtype mice had significantly lower levels of tau oligomers when compared to Control IgG (*p* = 0.0041), but did not show any differences with TOMA-treated A53T mice.

### TOMA treatment is associated with alterations in α-synuclein aggregation

**Fig. 4:**
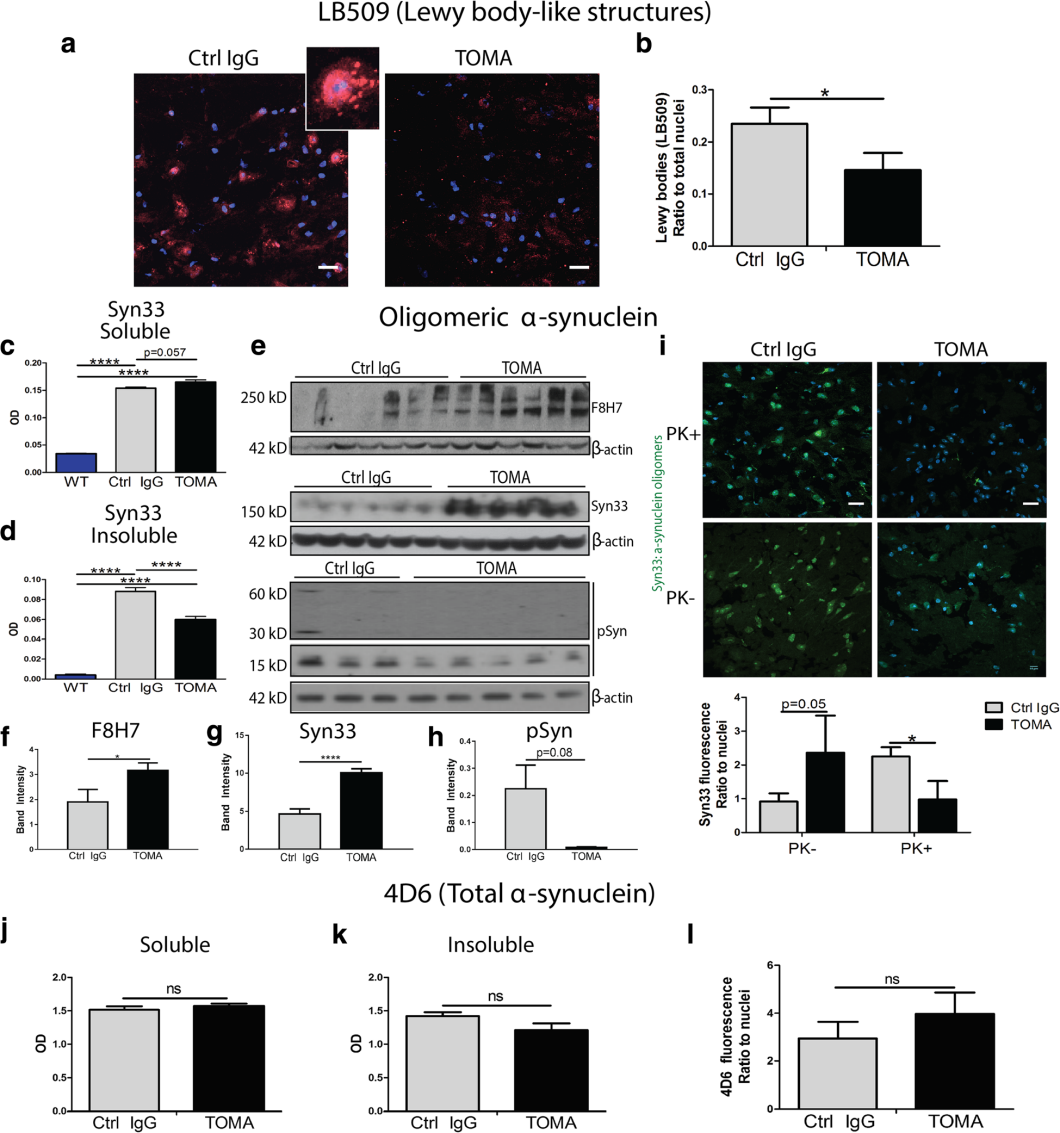
Lewy body-like structures are significantly decreased in TOMA-treated A53T mice. **a** α-synuclein structures were detected by immunofluorescence with LB509 (red) and nuclei were labeled with DAPI (blue). **b** Control IgG-treated mice had a significantly higher ratio of Lewy body-like structures to total nuclei when compared to TOMA-treated mice (*p* = 0.039). **c** Levels of soluble α-synuclein oligomers detected by ELISA with Syn33 were significantly elevated in both Control IgG and TOMA-treated A53T mice compared to wildtype mice (*p* < 0.0001) **d** PBS insoluble oligomeric α-synuclein was significantly increased in A53T mice treated with TOMA and Control IgG (p < 0.0001), however TOMA-treated mice had significantly decreased α-synuclein oligomers compared to Control IgG mice (p < 0.0001). **e** TOMA-treated mice had significantly higher soluble α-synuclein oligomers detected with (**f**) F8H7 (**g**) and Syn33 and (**h**) a trend toward lower phosphorylated α-synuclein by Western blot. **i** TOMA-treated mice showed elevated levels of α-synuclein oligomers detected with Syn33 over Control IgG approaching significance (*p* = 0.059; left panel), but PK-resistant α-synuclein oligomers were found to be significantly increased in Control IgG mice compared to TOMA (p = 0.036; right panel). **j**-**k** Levels of total α-synuclein detected by ELISA with 4D6 were unchanged in both soluble and insoluble fractions of total brain homogenate. **l** No differences were detected in total α-synuclein levels measured by fluorescence intensity (ratio to nuclei) with 4D6 between TOMA and Control IgG treatment groups. Scale bar 20 μm. TOMA (*n* = 6), Wildtype (*n* = 6), Ctrl IgG (*n* = 8)

In order to determine the effects of TOMA treatment on α-synuclein pathology, brain sections were analyzed with LB509 antibody. The ratio of Lewy body-like structures to total nuclei was quantified using immunofluorescence imaging (Fig. 4a-b). Control IgG-treated mice had a significantly higher ratio of Lewy body-like structures when compared to TOMA-treated mice (*p* = 0.039). In order to evaluate the effect of TOMA treatment on α-synuclein oligomers, which have been shown to cross-seed and co-aggregate with oligomeric tau, ELISA with Syn33 in both the PBS soluble and insoluble fractions of total brain homogenate was completed. As expected, soluble α-synuclein oligomers were significantly elevated in A53T mice when compared to wildtype mice (*p* < 0.0001); however, surprisingly, oligomeric α-synuclein was not decreased in the brains of TOMA-treated A53T mice compared to control IgG mice (Fig. 4c). Insoluble α-synuclein oligomers were again significantly elevated in A53T brain when compared to wildtype (*p* < 0.0001), but were also found to be significantly decreased in TOMA-treated mice compared to control IgG (*p* < 0.0001; Fig. 4d). PBS-soluble α-synuclein oligomers measured by Western blot with conformation-specific antibodies, F8H7 and Syn33, were significantly increased in TOMA-treated A53T mice compared to Control IgG (Fig. 4e-g). However, phosphorylated α-synuclein is more highly associated with later aggregation states, indicative of a shift toward the formation of Lewy body-like structures. Similar to results seen for decreased insoluble oligomers measured by ELISA, the phosphorylated α-synuclein oligomers trended toward a decrease in TOMA-treated mice compared to Control IgG (Fig. 4e, h). Previous study of α-synuclein toxicity has indicated that oligomers that are partially Proteinase K (PK)-resistant are the most toxic form of the protein [57]. Thus, immunofluorescence of brain sections with Syn33 was completed with and without PK treatment and α-synuclein oligomers were not decreased in the brain sections of TOMA-treated mice that were not treated with PK (Fig. 4i). However, proteinase K treatment showed that PK-resistant α-synuclein oligomers were significantly increased in Control IgG-treated mice compared to TOMA-treated mice (*p* = 0.036; Fig. 4i). In order to confirm that total α-synuclein levels were not altered by antibody treatment, ELISA with 4D6 antibody in both the PBS soluble and insoluble fractions of total brain homogenate was completed and no differences were found between groups (Fig. 4j-k). Additionally, measurement of fluorescence corrected for number of nuclei in sections labeled with 4D6 did not detect any differences between TOMA and Control IgG groups (Fig. 4l).

### TOMA treatment protects against loss of synaptic proteins and olfactory bulb dopamine

**Fig. 5:**
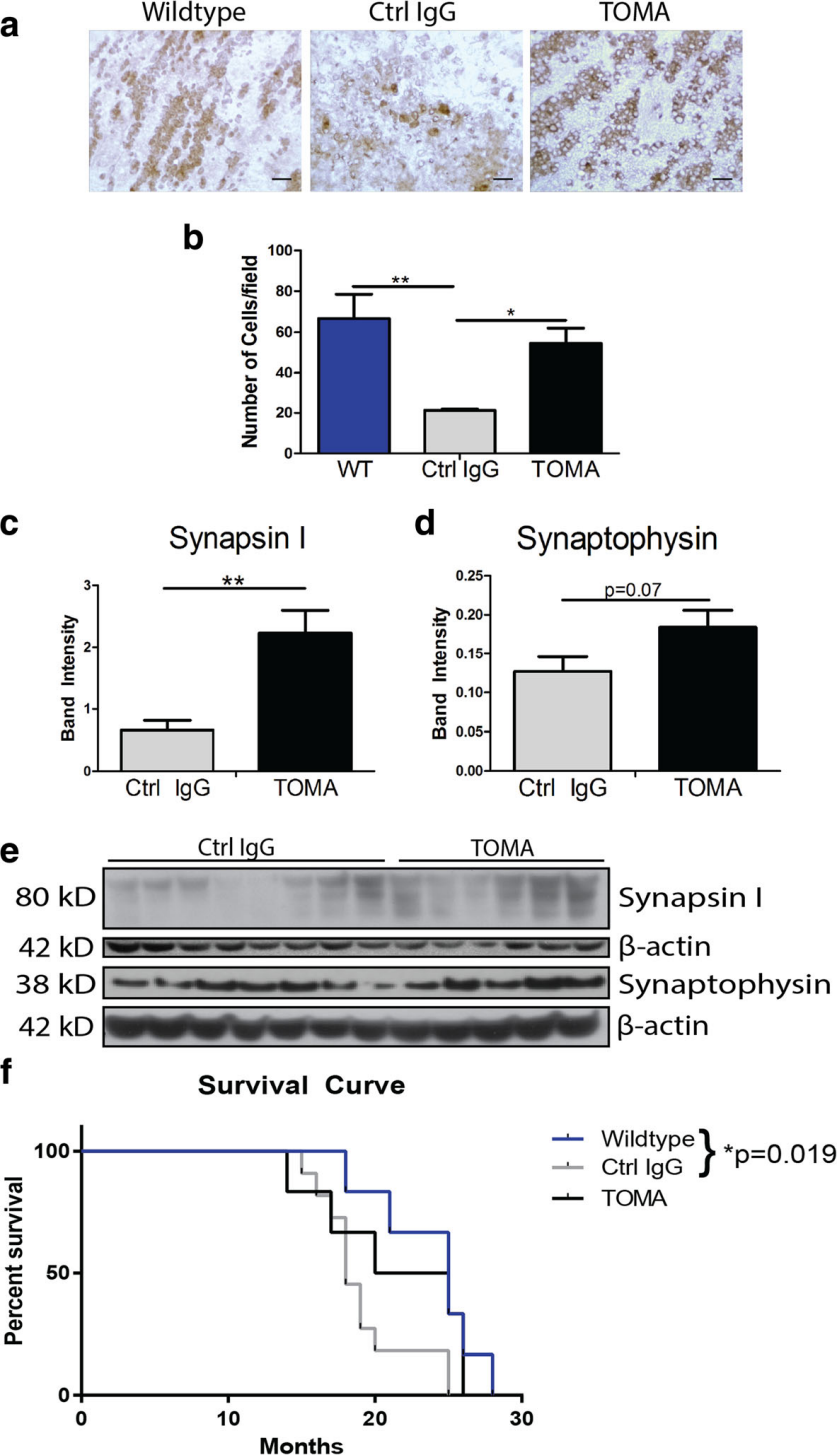
TOMA-treated mice are protected against dopamine loss in the olfactory bulb, synaptic dysfunction and premature death. **a** Representative images of olfactory bulb labeled with Tyrosine Hydroxylase (TH). **b** Number of TH-positive cells were significantly decreased in Control IgG-treated mice when compared to Wildtype (*p* = 0.001). TOMA-treated mice did not have significantly different levels of TH to Wildtype, but were significantly increased compared to Control IgG mice (*p* = 0.028). **c** and **e** Levels of synapsin 1 detected in the PBS soluble fraction of total brain homogenate by Western blot are significantly increased in TOMA-treated mice when compared to Control IgG (*p* = 0.003). **d-e** Synaptophysin levels are elevated in TOMA-treated mice approaching significance compared to control (*p* = 0.07). **f** Kaplan–Meier Survival Curves comparing Wildtype, Control IgG A53T and TOMA A53T mice. Control IgG-treated mice demonstrate a statistically significant (*p* = 0.019) increase in premature death rate when compared to Wildtype mice, while TOMA mice are not significantly different from either group. Scale bar 20 μm

In order to evaluate effects of antibody treatment on Parkinson’s disease-like toxicity, levels of catecholamines were measured by immunohistochemistry with Tyrosine Hydroxylase (TH) in the olfactory bulb, substantia nigra, striatum and locus coeruleus. Olfaction deficits are known to appear early in the course of disease in PD and are associated with a loss of dopamine in the olfactory bulb, as has also been seen previously in A53T mice [58–62]. While no differences were seen in the substantia nigra, striatum, or locus coeruleus (data not shown), Control IgG-treated mice had significantly decreased levels of the cells positive for the dopamine precursor when compared to Wildtype in the olfactory bulb (*p* = 0.001). TOMA-treated mice did not have significantly different levels of TH compared to Wildtype mice, but were significantly increased compared to control IgG mice (*p* = 0.028; Fig. 5a-b).

Rescue of synaptic toxicity was measured by Western blot with antibodies specific to synaptic proteins. TOMA treatment was associated with significantly elevated levels of synapsin 1 in the PBS soluble fraction of total brain homogenate when compared to Control IgG (*p* = 0.003; Fig. 5c, e) and a trend toward elevated levels of Synaptophysin (*p* = 0.07; Fig. 5d-e).

### TOMA treatment does not show signs of toxicity

In order to assess potential effects of TOMA treatment on survival, half of the treated mice in each group were assessed for mortality using Kaplan-Meier survival curves (Fig. 5f). A significant difference in survival was found between Wildtype and control IgG A53T mice (*p* = 0.019, Log-rank Mantel-Cox test with Bonferroni adjustment for multiple comparisons), while TOMA-treated mice did not show differences to Wildtype mice.

## Discussion

Synucleinopathies, including PD and DLB, have no effective treatment and affect millions of people worldwide. Most therapeutic approaches, thus far, have focused on targeting the symptoms or decreasing α-synuclein protein. Several studies have found evidence for α-synuclein oligomers as the most toxic form of the protein. Mouse models of synucleinopathy show deficits independent of the formation of fibrillar deposits and oligomeric α-synuclein induces toxicity in cell culture models, while fibrils do not [14–17]. However, research from our laboratory and others suggests that tau protein may also have a critical pathological role in synucleinopathies. We recently showed that the most toxic form of both tau and α-synuclein, oligomers, coexist and coaggregate in PD and DLB [41]. These results combined with studies showing a genetic and molecular interaction between tau and α-synuclein [26, 36–38] and the ability of α-synuclein oligomers to seed the aggregation of tau [27] suggest that the two proteins may have a synergistic interaction in disease. Therefore, targeting tau oligomers may prevent toxicity induced by tau, as well as affect synergy and co-aggregation with α-synuclein.

In order to inhibit toxicity of tau oligomers, we previously created a tau oligomer-specific antibody, TOMA, which we found to be effective in attenuating the disease phenotype in three different mouse models of tauopathy and Alzheimer’s disease [54, 55]. Importantly, in a model of Alzheimer’s disease with both Aβ and tau pathology, decreasing tau oligomers was not only effective at altering cognitive and synaptic dysfunction, but also shifted toxic oligomeric Aβ to a non-toxic conformation [54]. These results, combined with a number of other studies suggesting that Aβ triggers tau toxicity [63] support the hypothesis that targeting toxic tau may also rid the brain of toxic upstream targets, such as α-synuclein. Thus far, no studies have investigated the therapeutic potential of reducing tau oligomers in a synucleinopathy model. Here, we passively immunized a well-characterized synucleinopathy mouse model, the Prnp-SNCA*A53T model, which has been studied extensively and shown to develop insoluble α-synuclein aggregates, α-synuclein oligomers and hyperphosphorylated tau [26, 64–66]. The behavioral and pathological improvements seen in mice treated with TOMA suggested that lowering levels of tau oligomers may be sufficient to reduce toxicity due to tau and possibly α-synuclein toxicity in synucleinopathies. As levels of functional tau monomer were not found to differ between groups, differences due to loss of tau function between groups is likely minimal.

The co-aggregation and interaction of oligomeric tau and α-synuclein [41] led to the hypothesis that decreasing tau oligomers will also deplete oligomeric α-synuclein. However, surprisingly we found that decreases in α-synuclein oligomers by TOMA were detected only in α-synuclein oligomers with fibril-like characteristics, including insoluble, phosphorylated and proteinase-K (PK)-resistant oligomers. As a recent study showed that the most toxic form of oligomeric α-synuclein is partially resistant to PK [57], our results suggest that higher toxicity oligomeric α-synuclein structures were altered following depletion of tau oligomers. Thus, the relationship between tau oligomer depletion and α-synuclein aggregation appears to be multi-faceted. While TOMA treatment did not decrease total α-synuclein oligomer levels, the insoluble and proteinase-resistant form was lowered, as were Lewy body-like structures. It is possible that targeting tau interacting with α-synuclein alters its conformation or that changes may be dependent on timing. More study is warranted to further interrogate the synergy between the two aggregated proteins, however, beneficial effects due to tau oligomer depletion may initially occur independently of α-synuclein oligomer decline as tau toxicity lies downstream of α-synuclein. Thus, targeting tau alone could eliminate some toxicity without affecting upstream α-synuclein. The most effective treatment throughout time will likely require a combination of therapeutics against aggregates of both proteins.

While the effect of A53T expression in mice on dopamine levels has not been conclusively determined, TH levels in the olfactory bulb are decreased in A53T mice [58, 59]. Olfactory dysfunction and decreased dopamine has also been shown to be an important factor in both Alzheimer’s and PD [60–62]. Changes to TH levels may reflect a combination of both dopaminergic and noradrenergic effects, both of which may induce behavioral deficits in mouse models of synucleinopathy. Our results showing regulation of TH levels in the olfactory bulb by TOMA suggest that tau oligomer toxicity may impact dopamine and possibly other catecholamine levels.

## Conclusions

**Fig. 6:**
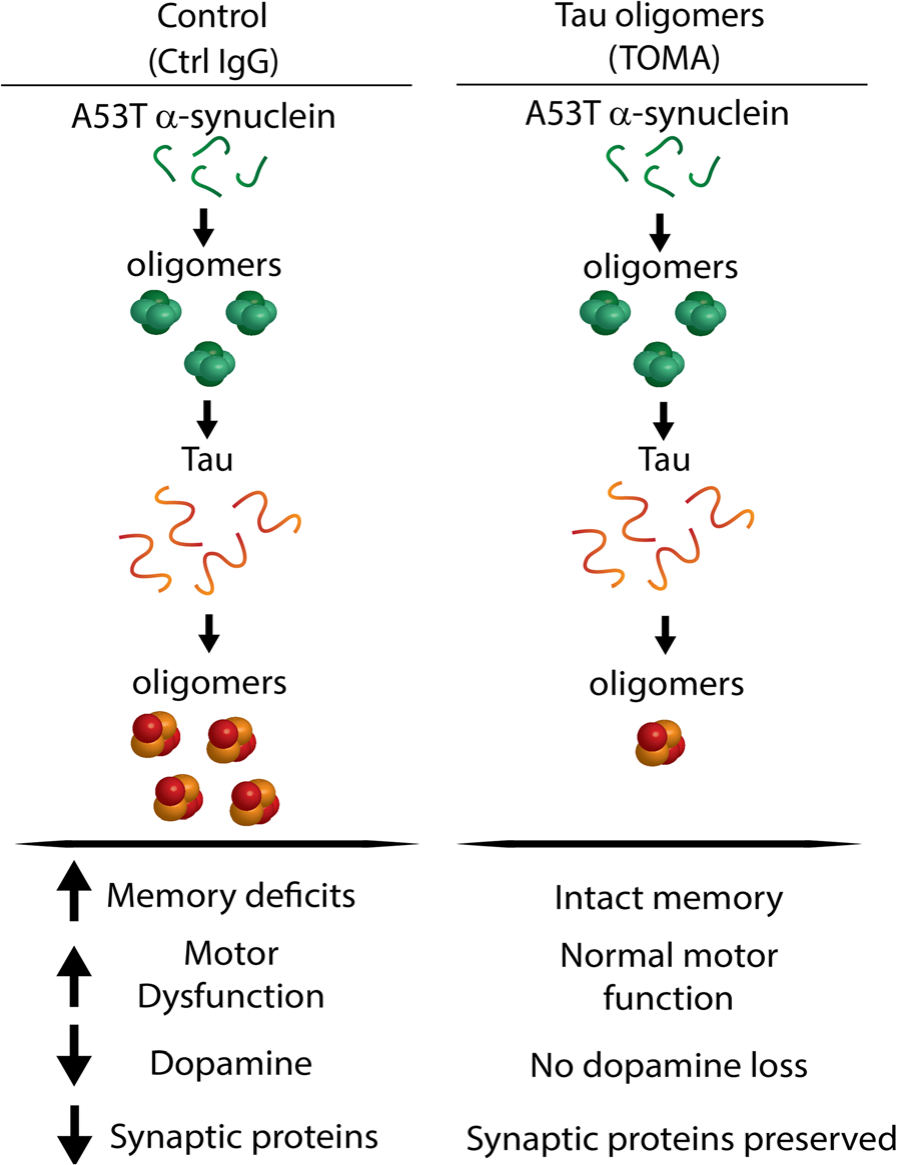
Schematic of the hypothesized interaction between upstream α-synuclein and downstream tau toxicity. Oligomeric α-synuclein cross-seeds tau, inducing its oligomerization, leading to downstream toxicity that is inhibited by immunotherapy against tau oligomers

Our results show for the first time, a benefit of a tau oligomer therapeutic for the prevention of cognitive and motor deficits, as well as brain pathology and synaptic dysfunction in a synucleinopathy model, suggesting that tau may be heavily involved in α-synuclein-related neurodegeneration (Fig. 6). Surprisingly, treatment with a tau oligomer-specific antibody did not decrease soluble α-synuclein oligomers broadly, though proteinase-resistant forms were lowered with treatment, as well as Lewy body-like structures. Further research will be necessary to conclusively determine the effects of depleting tau oligomers on α-synuclein aggregation. However, these findings suggest that a combination therapeutic approach using an antibody specific for oligomeric α-synuclein as well as tau, rather than either alone, may be the most effective strategy at reversing toxicity from both tau and α-synuclein oligomers.

## Abbreviations

AD: Alzheimer’s disease
ANOVA: Analysis of variance
Ctrl IgG: Control treatment group
HFIP: Hexafluoroisopropanol
HPLC: High-performance liquid chromatography
KO: Knockout
LB: Lewy body
LBD: Lewy body dementia
NFT: Neurofibrillary tangle
NOR: Novel object recognition
OCT: Optimal cutting temperature medium
PD: Parkinson’s disease
PK: Proteinase K
TH: Tyrosine hydroxylase
TOMA: Tau oligomer-specific monoclonal antibody
WT: Wildtype
